# Size and temperature effects on the viscosity of water inside carbon nanotubes

**DOI:** 10.1186/1556-276X-6-87

**Published:** 2011-01-17

**Authors:** Hongfei Ye, Hongwu Zhang, Zhongqiang Zhang, Yonggang Zheng

**Affiliations:** 1 State Key Laboratory of Structural Analysis for Industrial Equipment, Department of Engineering Mechanics, Faculty of Vehicle Engineering and Mechanics, Dalian University of Technology, Dalian 116023, China; 2 Center of Micro/Nano Science and Technology, Jiangsu University, Zhenjiang 212013, China

## Abstract

The influences of the diameter (size) of single-walled carbon nanotubes (SWCNTs) and the temperature on the viscosity of water confined in SWCNTs are investigated by an "Eyring-MD" (molecular dynamics) method. The results suggest that the relative viscosity of the confined water increases with increasing diameter and temperature, whereas the size-dependent trend of the relative viscosity is almost independent of the temperature. Based on the computational results, a fitting formula is proposed to calculate the size- and temperature- dependent water viscosity, which is useful for the computation on the nanoflow. To demonstrate the rationality of the calculated relative viscosity, the relative amount of the hydrogen bonds of water confined in SWCNTs is also computed. The results of the relative amount of the hydrogen bonds exhibit similar profiles with the curves of the relative viscosity. The present results should be instructive for understanding the coupling effect of the size and the temperature at the nanoscale.

## Introduction

Water conduction through single-walled carbon nanotubes (SWCNTs) has been paid much attention in recent years [1-5]. It is a significant topic for studying and designing the nanodevices such as the nanochannel for drug delivery and the membrane for water desalination [6-8]. The previous studies have revealed that the flow behavior of water at the nanoscale strongly depends on the characteristic length of nanochannel [9-12], which implies that the classical continuum theory for the macroscopic fluid may be no longer applicable for the fluid confined in nanochannels. Hence, many researches focused on the unique feature of the confined fluid and its relationship with the continuum fluid [9-13]. In classical continuum theory, the viscosity is an essential transport property and thereby has been extensively measured and computed [14,15]. The previous results have identified that the water viscosity relies on the temperature and the characteristic length of the nanochannel [9,12-15]. So far, the viscosity of fluids in nanoconfinement on a scale comparable to the molecular diameter is seldom explored owing to the extremely small scale on which the transport properties are difficult to be captured by experiments and the intrinsic limitations of the existing computational methods in the MD simulations [16-18]. This restricts the application of the classical continuum theory to the nanoflows.

Recently, an "Eyring-MD" method was proposed to calculate the viscosity of water by using the MD simulations [18]. In this article, we redetermine the coefficients in the "Eyring-MD" method through more numerical experiments and evaluate the viscosity of water inside SWCNTs at 298, 325, and 350 K. The objective of this study is to examine the size and the temperature effects on the water viscosity. Here, the size effect on the viscosity of the confined water implies the influence of the diameter of SWCNTs.

## The computational method

In the light of the "Eyring-MD" method, the viscosity *η *can be calculated by

(1) $$\eta =\frac{Nh}{V}\{\begin{array}{c} \exp\left[\frac{\sqrt{2\pi }{\text{(}E_{\text{c}}-\bar{E}\text{)}}^{2}+g_{2}\text{(}E_{\text{c}}-\bar{E}\text{)}\sigma +2g_{1}\sigma^{2}}{RT\sqrt{2\pi }\text{(}E_{\text{c}}-\bar{E}+g_{1}\sigma \text{)}}\right],\;E_{\text{c}}>\bar{E}\\ \exp\left\{1/RT\left[\begin{array}{l} \frac{\sqrt{2\pi }}{\sigma }\exp\left(\frac{{\left(E_{\text{c}}-\bar{E}\right)}^{2}}{2\sigma^{2}}\right)\\ -\frac{\sqrt{2\pi }\left(\bar{E}-E_{\text{c}}+g_{1}\sigma \right)}{\sqrt{2\pi }{\left(E_{\text{c}}-\bar{E}\right)}^{2}-g_{2}\left(E_{\text{c}}-\bar{E}\right)\sigma +2g_{1}\sigma^{2}} \end{array}\right]\right\},\;E_{\text{c}}\leq \bar{E} \end{array}$$

where *N *is the Avogadro's number, *h *is the Planck constant, *V *is the molar volume, *R *is the gas constant, *T *is the temperature, *g*_1 _= 3.333, and *g*_2 _= 7.32. $\bar{E}$ and *σ *are the average and the standard deviation of the potential energy occupied by the water molecules, respectively, which can be obtained by the MD simulations. *E*_c _is the critical energy and can be expressed as

(2) $$E_{\text{c}}=(aT+b)\sigma +(cT+d)+e\Delta U_{\text{coul}}$$

where the coefficients *a *= -0.001889 K^-1^, *b *= -1.232434, *c *= 0.017531 kcal mol^-1 ^K^-1^, *d *= -11.052943 kcal mol^-1^, and *e *= 0.56 are determined on the basis of the previous numerical experiments of the bulk water at 298 and 350 K and the new numerical experiments at 325 K. The last term in Equation 2 is a correction term, in which Δ*U*_coul _can be calculated by

(3) $$\Delta U_{\text{coul}}=U_{\text{coul}}-f_{1}U_{\text{van}}-f_{2}$$

in which *U*_coul _and *U*_van _are the coulomb energy and the van der Waals energy extracted from the MD simulations. The coefficients *f*_1 _= -2.062576 and *f*_2 _= -8.984223 kcal mol^-1 ^at 298 K, *f*_1 _= -2.058061 and *f*_*2 *_= -8.742694 kcal mol^-1 ^at 325 K, and *f*_1 _= -2.065280 and *f*_2 _= -8.502127 kcal mol^-1 ^at 350 K. Thus, by using Equations 1, 2, and 3, the viscosity of water can be predicted by the MD simulations. The correlation coefficient between the viscosity calculated by the "Eyring-MD" method and that obtained from the numerical experiments (Stokes-Einstein relation) is about 0.99.

**Figure 1:**
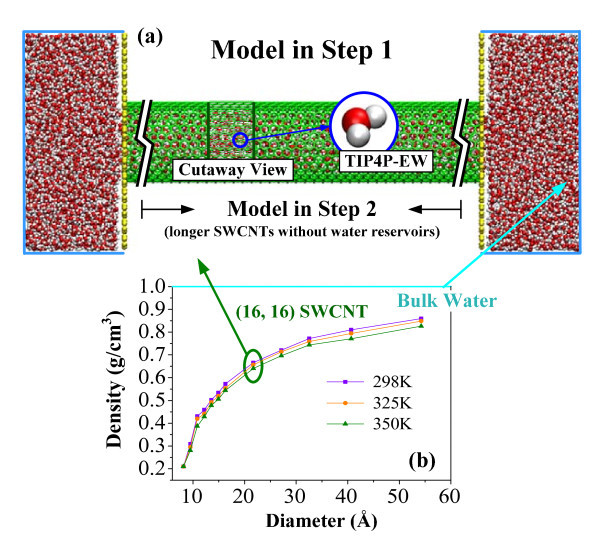
**The computational models in the MD simulations**. **(a) **The MD models for the (16, 16) SWCNT; **(b) **the density of the confined water against the diameter.

In this article, an open-source code Lammps is employed to conduct the MD simulations [19]. The MD models are depicted in Figure 1a. To save the computational cost, the carbon atoms of the SWCNTs and the graphite sheets are fixed. The water is simulated by the TIP4P-EW model [20], in which the SHAKE algorithm is used to constrain the bond length and angle of the water molecules. The interactions between the carbon atoms and the oxygen atoms of the water molecules are calculated by the Lennard-Jones (LJ) potential with the main parameters *σ*_CO _= 3.28218 Å and *ε*_CO _= 0.11831 kcal mol^-1^. The periodic boundary condition is applied to all the three directions of the three-dimensional simulation system. The cutoff distances for the LJ interactions and the electronic interactions are 10 and 12 Å, respectively. The particle-particle particle-mesh algorithm is adopted to handle the long-range coulomb interactions. To examine the size effect on the water viscosity, we consider the armchair SWCNTs of diameter in a wide range from 8 Å ((6, 6) SWCNT) to 54 Å ((40, 40) SWCNT). The simulation is performed in the NVT ensemble with the integral time step of 1 fs and can be divided into two steps. First, a SWCNT (60 Å in length) and two water reservoirs are equilibrated for 80 ps, during which the density of the water in the reservoirs away from the tube entrances is maintained constant at different temperatures (0.99 g/cm^3 ^at 298 K, 0.98 g/cm^3 ^at 325 K, and 0.96 g/cm^3 ^at 350 K). The purpose is to calculate the density of water inside various SWCNTs, as shown in Figure 1b. Then, the two reservoirs are removed and a longer SWCNT is adopted as the second model to equilibrate for 600 ps, and the data are collected within the last 500 ps. The length of the SWCNTs in this step is so long that enough water molecules (more than 860) can be contained. The above two-step simulation focuses all the computational consumption on the concerned information.

## Results and discussion

**Figure 2:**
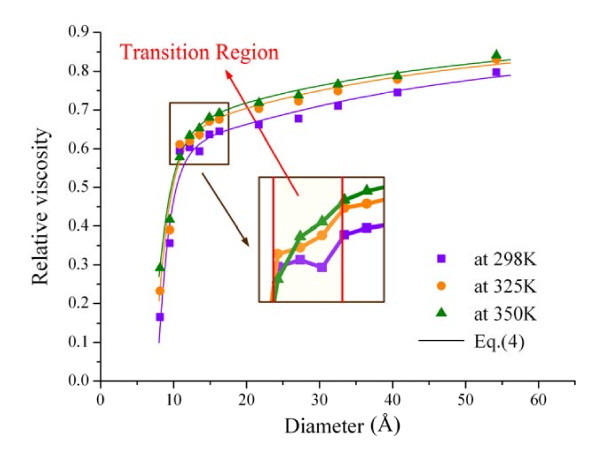
**The variations of the relative viscosity of water confined in SWCNTs with the diameter**.

Figure 2 shows the relative viscosity of water confined in SWCNTs versus the diameter at 298, 325, and 350 K. The relative viscosity is the ratio of the viscosity of the confined water to the viscosity of the bulk water, i.e., *η*_*r *_= *η*_cnt_/*η*_bulk_. Here, the viscosities of the bulk water at the three temperatures are 0.668 mPa s at 298 K, 0.426 mPa s at 325 K, and 0.307 mPa s at 350 K, respectively. The adoption of the relative viscosity makes the comparison of the size dependences of the relative viscosity at different temperatures clearer. From Figure 2, it can be seen that the size-dependent trends of the relative viscosity at the three temperatures are similar. For a specified diameter, the relative viscosity increases with increasing temperature, and the increasing extent nonlinearly varies with the diameter of SWCNTs. For a specified temperature, the relative viscosity of water confined in SWCNTs increases with enlarging diameter of SWCNTs. When the diameter is lower than 10.5 Å, the relative viscosity dramatically increases with the diameter. For the diameter varying from 10.5 to 14.5 Å, the relative viscosity is in a transition state from the sharp variation to a smooth region (see the transition region in Figure 2). As the diameter further increases, the curves gradually flatten and approach 1.0, which is the relative viscosity of the bulk water.

**Figure 3:**
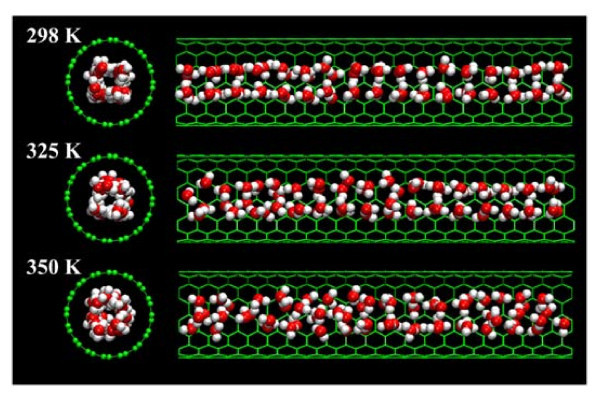
**The snapshots of the configurations of the water molecules inside the (8, 8) SWCNT at 298, 325, and 350 K**.

Furthermore, from the inset in Figure 2, some anomalous increments can be detected in the relative viscosity inside the SWCNTs of diameter ranging from 10.5 Å to 14.5 Å at 298 and 325 K. These increments in the transition region can be ascribed to the structural configuration of the water molecules inside the (8, 8) and (9, 9) SWCNTs. Figure 3 presents the configurations of the water molecules inside the (8, 8) SWCNT at 298, 325, and 350 K. It can be seen that the water molecules exhibit a hollow, close, and ordered arrangements at 298 K, which could enhance the combinations among the water molecules and result in an increment in the relative viscosity. As the temperature increases, this structural configuration gradually disappears since the thermal motions of the water molecules get faster, which can associate with the disappearance of the anomalous increments of the relative viscosity at 350 K. Hence, the changes in the configuration can well explain the anomalous increments of the relative viscosity in the transition region. Furthermore, it should be noted that the structural configuration of the water molecules is similar to the molecular configuration of ice whose viscosity is underestimated by the "Eyring-MD" method [18]. Nevertheless, the present predictions for the viscosity at 298 and 325 K in the transition region should be still acceptable because the water is not yet ice in this case [21,22].

According to the calculated results, a formula of the water viscosity is fitted as follows:

(4) $$\eta =\eta_{\text{bulk}}\left[1-{\left(\frac{r_{1}}{d}\right)}^{c_{1}}+{\left(\frac{r_{21}T+r_{22}}{d}\right)}^{c_{2}}-{\left(\frac{r_{31}T+r_{32}}{d}\right)}^{c_{3}}\right]$$

in which *d *is the diameter of SWCNTs, *T *is the temperature, *r *represents the fitting coefficients: *r*_1 _= 5.2 Å, *r*_21 _= -0.004506 Å/K, *r*_22 _= 10.710977 Å, *r*_31 _= -0.007179 Å/K, *r*_32 _= 11.275373 Å, the viscosity of the bulk water *η*_bulk_, and the exponentials *c *are expressed as:

(5) $$\begin{array}{l} \eta_{bulk}=p_{1}\exp(p_{2}/T)\\ c_{1}=(p_{11}T+p_{12})\exp(p_{13}/T)\\ c_{2}=p_{21}T+p_{22}\\ c_{3}=p_{31}T+p_{32} \end{array}$$

where *p*_1 _= 0.00285 mPa s, *p*_2 _= 1632 K, *p*_11 _= 0.000225 1/K, *p*_12 _= -0.055547, *p*_13 _= 1197.417113 K, *p*_21 _= -0.007639 1/K, *p*_22 _= 4.910991, *p*_31 _= -0.011533 1/K, and *p*_32 _= 7.240463. The computational results of Equation 4 are also displayed in Figure 2 (lines). The correlation coefficient between the fitting results (lines in Figure 2) and the relative viscosity (symbols in Figure 2) is about 0.96. Furthermore, it should be noted that the *η*_bulk _in Equation 5 calculates the temperature-dependent viscosity of the bulk water, which is fitted according to the widely accepted exponential relationship [23] and the viscosities of bulk water within the temperature range from 275 to 400 K from the MD simulations. This term will become dominant when the size (*d*) gradually tends to infinite, which is consistent with the physical role of the confinement. Equation 4 describes the size and the temperature effects on the water viscosity and should be significant for the research on the flow behavior at the nanoscale.

**Figure 4:**
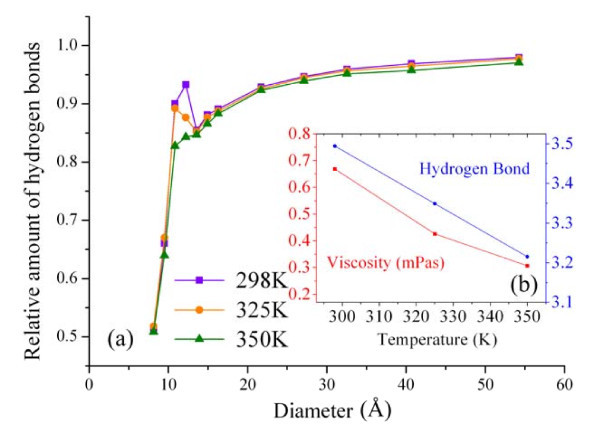
**The hydrogen bond of water**. **(a) **The relative amount of the hydrogen bonds of the confined water versus the diameter; **(b) **the comparison of the amount of the hydrogen bonds and the viscosity of the bulk water at the three temperatures.

To further understand the size and the temperature influences, the amount of the hydrogen bonds of water confined in SWCNTs is also studied. The amount of the hydrogen bonds can be used to characterize the stability of the microstructure of water molecules [1,24]. In general, a larger amount of the hydrogen bonds implies stronger intermolecular interactions among the water molecules, which could result in an increase in the viscosity. This qualitative relation can be drawn from Figure 4b and utilized to verify the predictions of the relative viscosity. Figure 4a illustrates the variation of the relative amount of the hydrogen bonds of water confined in SWCNTs with the diameter. The relative amount is the ratio of the amount of the hydrogen bonds of the confined water to the amount in the bulk water. In this study, the geometrical definition of the hydrogen bond is adopted [25]. The amounts of the hydrogen bonds of the bulk water are 3.494 at 298 K, 3.349 at 325 K, and 3.215 at 350 K. From Figure 4a, it can be seen that the relative amount of the hydrogen bonds exhibits a similar trend with the relative viscosity. In the transition region, some remarkable increments can be found in the relative amounts of the hydrogen bonds at 298 and 325 K, which are also consistent with the anomalous increments in the relative viscosity. While for a given diameter, the relative amount of the hydrogen bonds slightly decreases with increasing temperature, which is in contrast to the trend of the relative viscosity. This inconsistency can be ascribed to the different temperature-dependent trends of the viscosity (nonlinear) and the hydrogen bond (linear) of the bulk water, as shown in Figure 4b.

## Conclusions

In summary, we have studied the influences of the diameter of SWCNTs and the temperature on the viscosity of the confined water by using the "Eyring-MD" method whose coefficients are redetermined through considering new numerical experiments. For a specified temperature, the relative viscosity nonlinearly increases with enlarging diameter of SWCNTs. For a given diameter, the relative viscosity of water inside the SWCNTs increases with increasing temperature. An approximate formula of the relative viscosity with consideration of the size and the temperature effects is proposed, which can avoid the time-consuming MD simulations and should be significant for the research on the water flow inside the nanochannels. Furthermore, the amount of the hydrogen bonds of water confined in SWCNTs is also computed. The results suggest that the relative amount of the hydrogen bonds has similar profile with the relative viscosity, which demonstrates the present predictions of the relative viscosity. The computations in this study reveal that the trend of the size dependence is almost insensitive to the temperature, whereas the size-dependent extent could vary with the temperature. This finding provides an insight into the researches on the nanoflows and is instructive for understanding the coupling effect of the size and the temperature at the nanoscale.

## Abbreviations

LJ: Lennard-Jones; MD: molecular dynamics; SWCNTs: single-walled carbon nanotubes.

## Competing interests

The authors declare that they have no competing interests.

## Authors contributions

HZ and HY conceived and designed this work. HY and ZZ performed the MD simulations. HY, YZ and ZZ collected and analyzed the data. All authors discussed the results and edited the manuscript. All authors read and approved the final manuscript.
